# rLj-RGD3, a Novel Recombinant Toxin Protein from *Lampetra japonica*, Protects against Cerebral Reperfusion Injury Following Middle Cerebral Artery Occlusion Involving the Integrin-PI3K/Akt Pathway in Rats

**DOI:** 10.1371/journal.pone.0165093

**Published:** 2016-10-21

**Authors:** Qian Lu, Jihong Wang, Junshu Jiang, Shengnan Wang, Qilan Jia, Yue Wang, Weiping Li, Qin Zhou, Li Lv, Qingwei Li

**Affiliations:** 1 Department of Pharmacology, Dalian Medical University, Dalian, Liaoning Province, China, 116044; 2 School of Life Sciences, Liaoning Normal University, Dalian, Liaoning Province, China, 116029; 3 Key Laboratory of Biotechnology and Drug Discovery of Liaoning Province, Dalian, Liaoning Province, China, 116029; 4 College of Basic Medicine, Jilin Medical College, Jilin, Jilin Province, China, 132013; Universita degli Studi di Napoli Federico II, ITALY

## Abstract

**Background:**

The RGD-toxin protein Lj-RGD3 is a naturally occurring 118 amino acid peptide that can be obtained from the salivary gland of the *Lampetra japonica* fish. This unique peptide contains 3 RGD (Arg-Gly-Asp) motifs in its primary structure. Lj-RGD3 is available in recombinant form (rLj-RGD3) and can be produced in large quantities using DNA recombination techniques. The pharmacology of the three RGD motif-containing peptides has not been studied. This study investigated the protective effects of rLj-RGD3, a novel polypeptide, against ischemia/reperfusion-induced damage to the brain caused by middle cerebral artery occlusion (MCAO) in a rat stroke model. We also explored the mechanism by which rLj-RGD3 acts by measuring protein and mRNA expression levels, with an emphasis on the FAK and integrin-PI3K/Akt anti-apoptosis pathways.

**Methods:**

rLj-RGD3 was obtained from the buccal secretions of *Lampetra japonica* using gene recombination technology. Sprague Dawley (SD) rats were randomly divided into the following seven groups: a sham group; a vehicle-treated (VT) group; 100.0 μg·kg^-1^, 50.0 μg·kg^-1^ and 25.0 μg·kg^-1^ dose rLj-RGD3 groups; and two positive controls, including 1.5 mg·kg^-1^ Edaravone (ED) and 100.0 μg·kg^-1^ Eptifibatide (EP). MCAO was induced using a model consisting of 2 h of ischemia and 24 h of reperfusion. Behavioral changes were observed in the normal and operation groups after focal cerebral ischemia/reperfusion was applied. In addition, behavioral scores were evaluated at 4 and 24 h after reperfusion. Brain infarct volumes were determined based on 2,3,5-triphenyltetrazolium chloride (TTC) staining. Pathological changes in brain tissues were observed using hematoxylin and eosin (H&E) staining. Moreover, neuronal apoptosis was detected using terminal deoxynucleotidyl transferase-mediated dUTP-biotin nick-end labeling (TUNEL) assays. We determined the expression levels of focal adhesion kinase (FAK), phosphatidyl inositol 3-kinase (PI3K), protein kinase B (Akt, PKB), caspase-3 and Bcl-2 in the brain using western blot analysis and RT-PCR assays. The research protocol was approved by the Institutional Ethics Committee of Dalian Medical University.

**Results:**

The behavioral scores and cerebral infarct volumes of the rLj-RGD3 groups were markedly lower at 4 and 24 h/RF. The rLj-RGD3 protein significantly ameliorated pathological changes in the brain and reduced the number of apoptotic neurons. Furthermore, the FAK and PI3K/Akt pathways were activated. rLj-RGD3 significantly increased the expression of FAK, p-FAK and Bcl-2 proteins. In contrast, caspase-3 expression was inhibited.

**Conclusion/Significance:**

We conclude that recombinant *Lampetra japonica* RGD-peptide (rLj-RGD3) exerts a protective effect against cerebral ischemia/reperfusion injury in the brain. In addition, the mechanism of this protection is associated with the activation of the integrin-PI3K/Akt pathway. These results provide a theoretical foundation and an experimental basis for using RGD peptides as novel drugs for treating ischemic cerebral vascular diseases in addition to promoting the research and development of marine biotechnology drugs.

## Introduction

Pierschbacher and Ruoslahti [1] were the first to show, in 1984, that the RGD sequence serves as a binding site between fibronectin (FN) and its receptor. In 1985, Hayman et al. [2] demonstrated that peptides containing an RGD sequence can inhibit cell adhesion. Hynes [3] named the RGD receptor “integrin” in a 1987 summary of the biological properties of this molecule. Over the past 2 decades, our group has focused on creating recombinant Lj-RGD3 because it is difficult to obtain endogenous Lj-RGD3. In the early 2000s, we used DNA recombination techniques to produce recombinant Lj-RGD3 (rLj-RGD3) in large quantities for the first time.

Integrins mediate biological processes that involve interactions between cells and other cells or their substrates [4]. Integrin signal transduction regulates many processes, including cell cycle entry, apoptosis, gene transcription, pH adjustment, and cell migration [5–7]. In addition, these molecules participate in several physiological and pathological processes, including tumor vessel generation [8], invasion and metastasis [9,10], inflammation [11], and blood clotting [12,13]. The RGD sequence has been identified in 67 types of proteins in the body, including fibronectin (FN), laminin (LM), vitronectin, collagen and von Willebrand factor [14]. Many reports have described the effects of RGD-containing peptides, including their ability to limit cancer cell transfer, generate vascular endothelial cells, and aggregate platelets. Thus, the RGD sequence is considered a target for drug development. Reports [15] have described various structural modifications that affect RGD-containing peptides and their impact on diverse biological activities.

Cerebral ischemic disease seriously affects the health and daily life of affected patients [16] and is a major cause of death. When cerebral arterial atherosclerosis and thrombi form, the lumen of the artery can form a stenosis or even an occlusion, leading to the acute, focal restriction of blood supply and cerebral ischemia. Cerebrovascular embolism is a prevalent condition that accounts for 40–60% of this type of pathogenesis. Ischemic stroke accounts for approximately 85% of all strokes in the West and 70% of those in the Orient [17].

Recanalizing blood vessels in the brain might be a reasonable method for treating ischemia, although immediate reperfusion of an ischemic brain can have harmful consequences [18,19]. Treatments that not only inhibit blood aggregation but also protect the brain from the damage it can incur upon reperfusion are urgently needed. RGD-containing peptides have attracted increasing attention because they possess the ability to specifically bind surface integrins by competing with extracellular matrix proteins. However, few reports have examined the details of the mechanisms by which RGD-containing peptides protect the brain. The experiments presented in this paper were designed to investigate how rLj-RGD3 prevents cerebral lesions after ischemia/reperfusion in rats at the biochemical and molecular levels. The RGD-containing peptide Lj-RGD3 contains three RGD sequences and can be isolated from the oral secretions of *Lampetra japonica*. Recent developments have allowed us to produce a recombinant form of this peptide (designated rLj-RGD3) *in vitro* using gene recombination methods. We predict that rLj-RGD3 diminishes neurocyte apoptosis by activating the integrin-PI3K/AKT pathway and that this is a potential mechanism for preventing post-ischemic injury.

In this study, we used RNA that was isolated from the buccal gland secretions of *Lampetra japonica* to obtain rLj-RGD3 using recombinant DNA technology. We evaluated the neuroprotective effects of rLj-RGD3 against apoptosis in cerebral cortical neurons and against ischemia-induced brain damage and cell death in a rat model of middle cerebral artery occlusion/reperfusion. Our study is the first to demonstrate that the activation of the integrin-PI3K/AKT pathway is involved in the anti-apoptotic, brain cell damage-ameliorating effects of RGD-containing peptides.

## Materials and Methods

### Materials

The recombinant plasmid pET23b-RGD3 is stored in our laboratory. Histidine affinity chromatography columns (His. Bind Column) were purchased from Novagen. Tricine and isopropyl β-D-1-thiogalactopyranoside (IPTG) were purchased from Amresco.

### Methods

The research protocol was approved by the Institutional Ethics Committee of Dalian Medical University.

#### Extraction and purification of rLj-RGD3 protein

BL21 *Escherichia coli* (*E*. *coli*) cells containing recombinant plasmids were cultured until the cells reached the logarithmic phase of growth. Then, IPTG was added at a final concentration of 1 mM, and the cells were cultured at 30°C overnight to induce the expression of soluble rLj-RGD3. The cells were then sonicated and centrifuged, and the target proteins were purified using histidine affinity chromatography according to the manufacturer’s instructions.

The purified rLj-RGD3 proteins were identified using Tricine-SDS PAGE, during which they were subjected to sample loading, fixation, staining and destaining until clear protein bands were visible.

#### Establishing an injury model

Male Sprague Dawley (SD) rats weighing 280 to 320 g were housed under constant environmental conditions (temperature, 24±2°C; humidity, 55±5%; 12/12 h light/dark cycle) in the SPF Laboratory Animal Center at Dalian Medical University. The rats had free access to food and water before and after all procedures. Anesthesia was induced using 10% chloral hydrate (0.35 mg·kg^-1^) via intraperitoneal injection. During surgery, rectal temperature was monitored and maintained at 38.0±0.5°C. Focal cerebral ischemia was induced via middle cerebral artery occlusion (MCAO) [20] using disposable nylon filaments that were 0.26–0.32 mm in diameter. Briefly, the nylon monofilament was threaded through the external carotid artery to the internal carotid artery to block the middle cerebral artery. The monofilament was then removed to restore blood flow. Rats undergoing ischemic reperfusion recovered in their home cage. Transient focal cerebral ischemia was induced in the area that was perfused by the middle cerebral artery (MCA) [21]. Briefly, an incision was made at the midline of the neck to expose the right carotid bifurcation. The internal carotid artery (ICA) was followed rostrally, and the pterygopalatine branch was identified and ligated. The common carotid artery (CCA) was then occluded, and the branches of the external carotid artery (ECA) were dissected and divided. To occlude the closure, a 4–0 nylon suture with a silicone-coated tip was advanced from the ECA into the lumen of the ICA until it blocked the origin of the MCA. The average size of the silicone-coated portion was 0.26 mm in diameter and 30.0 mm in length. We chose an occlusion that blocked the MCA at a depth of 18–20 mm. The right middle cerebral artery was occluded for 2 h and then reperfused for 24 h. Carprofen (5–15 mg·kg^-1^ SQ q 24 h) was used as an analgesic. All rats were randomly assigned to one of seven experimental groups (n = 15 per group) as follows: (1) sham surgery without ischemia; (2) ischemia for 2 h and reperfusion for 24 h (vehicle-treated group); (3) ischemia plus postconditioning, rLj-RGD3 high (100.0 μg·kg^-1^) group; (4) ischemia plus postconditioning, rLj-RGD3 middle (50.0 μg·kg^-1^) group; (5) ischemia plus postconditioning, rLj-RGD3 low (25.0 μg·kg^-1^) group; (6) ischemia plus postconditioning, Edaravone 1.5 mg·kg^-1^ positive control group; and (7) ischemia plus postconditioning, Eptifibatide 100.0 μg·kg^-1^ positive control group. The drugs were administered intravenously 10 minutes after reperfusion. To further study the efficacy of the drugs, we also administered the drugs intravenously 5 hours after reperfusion and then analyzed ischemic damage and neurological function at 3 and 7 days after ischemia in an additional experiment. In the sham group, the rats were subjected to sham surgery without ischemia and injected with the same volume of normal saline as that administered via injection in the other groups. We used a protocol with humane endpoints to manage cases where rats became severely ill prior to the experimental endpoint. When the rats showed clinical signs, including severe respiratory depression, convulsions and fainting, they were euthanized. The method of euthanasia was decapitation after a sedative was injected. All of the rats (including the rats that were euthanized prior to the experimental endpoint) were euthanized.

#### Cell culture and drug treatments

Normal PC12 cells were purchased from the Institute of Cell Biology, Chinese Academy of Sciences (Shanghai) and maintained in Dulbecco’s Modified Eagle’s Medium (DMEM, Sigma, USA) supplemented with 5% heat-inactivated fetal calf serum (FCS, Sigma, USA), 10% heat-inactivated horse serum, penicillin (50 U/mL), and streptomycin (50 mg/L). PC12 cells were cultured at 37°C in a humidified atmosphere of 5% CO_2_ and 95% air.

PC12 cells were subjected to OGD-R as previously described [22]. Briefly, the original medium was removed, and the cells were washed with Earle’s balanced salt solution (EBSS) at pH 7.4 and placed in fresh glucose-free EBSS supplemented with Na_2_S_2_O_4_. The culture dishes were then introduced in a mixture of 5% CO_2_ and 95% air at 37°C for 2 h, after which the medium was replaced with fresh DMEM for another 24 h. The control culture was maintained in normal EBSS and incubated under normal conditions. The cells were divided into five groups: the control group; the OGD-R group; the OGD-R+rLj-RGD3 group, the OGD-R+MK-2206 (5 μM, Selleck, USA) group and the OGD-R+MK-2206+rLj-RGD3 group.

#### Neurological evaluation

Behavioral scores were measured at 4 and 24 h after reperfusion. A behavioral test was used to quantify the motor asymmetry that was caused by unilateral cortical stroke. The test was performed by a person who was blinded to the experimental conditions. Rats were handled for 3 days before stroke was induced, and the baseline was recorded on the day before surgery. Two to three persons who were blinded to the experimental conditions performed all required behavior tests for 24 h post-stroke. Scoring criteria were based on Bederson et al. [23] but were modified as follows. (1) The rats were placed on smooth ground, and each rat’s shoulders were pushed to the side to determine resistance. If bilateral resistance was equal and strong, the rat was scored as 0; the rats in which the resistance of the shoulder contralateral to the operated shoulder was lower were divided into light, medium and heavy grades and scored as 1, 2, and 3, respectively, according to the extent of the decline. (2) Forelimb buckling was analyzed as follows: if both forelimbs symmetrically reached towards the ground, the rat was scored as 0. The rats in which the forelimb contralateral to the operative forelimb showed wrist flexion, elbow flexion, shoulder rotation or all three symptoms were scored as 1, 2, 3, and 4, respectively. (3) Both forelimbs were placed onto a metal net to study muscle tension. If muscle tension was equal and strong in both forelimbs, the rat was scored as 0. Otherwise, the rats were scored as 1, 2, or 3 according to the extent of the observed decline in muscle tension. (4) If the rat maintained movement on the injured side, the rat was scored as 1. The highest score was 11, and higher scores indicated a more serious behavioral disorder.

#### Measurement of infarct size and general histology

After a sedative was injected, the rats were sacrificed using decapitation. The brain was immediately removed, and infarct volumes were determined using 2,3,5-triphenyltetrazolium chloride (TTC) staining. The brains were sectioned into five coronal blocks from rostral (level 1) to caudal (level 5) and then stained with a 2% TTC solution in culture plates. The plates were maintained at 37°C for 20–30 minutes. The percentage of the volume that was affected by the infarct was calculated according to the following formula: [contralateral volume—(ipsilateral volume—infarct volume) / contralateral volume] · 100%. Brain tissues were stored in a 4% methanol solution before pathological changes were detected using H&E staining.

#### Neuronal apoptosis

To screen for the presence of apoptotic cells, we observed neuronal apoptosis in brain tissue slices using a TUNEL Kit (Roche Ltd, Switzerland, Germany). Briefly, fresh brain tissue was paraffinized and cut into sections. Then, the tissue was soaked for 8 min in dimethylbenzene and PBS (0.01 M·L^-1^, pH 7.2: 8.0 g·L^-1^ NaCl, 0.2 g·L^-1^ KCl, 1.44 g·L^-1^ Na_2_HPO_4_·2H_2_O, and 0.24 g·L^-1^ KH_2_PO_4_) to dewax and hydrate the sections. Then, the sections were washed with PBS twice for three minutes each. Finally, premade TUNEL reaction solution was added to the surfaces of the brain tissue sections, which were maintained in a warm bath at 37°C for 90 min. Neuronal apoptosis was analyzed using optical microscopy.

#### Western blot analysis

Western blot analysis was performed as previously described using phospho-specific antibodies that were raised against protein kinases. Pallial tissue corresponding to the ischemic region was dissected. Whole cell proteins were extracted from the fresh brain tissue. Cells were collected after treatment, and whole cell lysates were prepared by incubation in RIPA buffer (Cell Signaling Technology, Boston, MA, USA) supplemented with a protease inhibitor cocktail (Roche), according to the manufacturer’s instructions. The lysates of cell protein and tissue protein (20 μg protein) were separated by sodium dodecyl sulfate–polyacrylamide gel electrophoresis (SDS-PAGE) (6%-12%) and transferred to polyvinylidene fluoride membranes. Bound antibodies were visualized using an ECL Advance western blotting detection kit (GE Healthcare, Little Chalfont, Buckinghamshire, UK), and images were obtained using a LAS-4000 imaging system (Fuji Film, Tokyo, Japan).

#### RT-PCR analysis

Total RNA was isolated from brain cortical tissues using TRIzol Reagent (KeyGEN BioTECH, China). RT-PCR was performed using TaqMan Gene Expression Assays (Applied Biosystems, USA) on an ABI-prism 7700 instrument. Two micrograms of total RNA were used to determine the mRNA level. FAK, PI3K, Bcl-2, and caspase-3 mRNA expression levels were normalized to GAPDH expression and analyzed relative to the appropriate controls. The following primers were used: PI3K (p85) forward, *5’-CAGATGCTTTCAAACGCTAT-3’* and reverse, *5’-GAAGTGGGCTTGGGTGGT-3’*; Bcl-2 forward, *5’-GGCATCTTCTCCTTCCAGC-3’* and reverse, *5’-CATCCCAGCCTCCGTTAT-3’*; caspase-3 forward, *5’-ATGTCGATGCAGCTAACC-3’* and reverse, *5’-GTCTCAATACCGCAGTCC-3’*; and GAPDH forward, *5’- TCAACGGCACAGTCAAGG-3’* and reverse, *5’-ACCAGTGGATGCAGGGAT-3’*. The resulting PCR products were subjected to 1.5% agarose gel electrophoresis. The genes of interest were quantified relative to GAPDH levels. A BioSpectrum-410 multispectral imaging system equipped with a Chemi HR camera 410 (UVP, Inc., Upland, CA, USA) was used to analyze the intensity of the electrophoresis bands.

#### Statistical analysis

For the infarctions and western blots, we performed one-way ANOVA followed by the Tukey test. All results were considered statistically significant at a p-value < 0.05. Data are presented as the means ± SD. All statistical analyses were performed using SPSS16 (SPSS, Inc., Chicago, IL, USA).

## Results

### Production of purified rLj-RGD3 protein

**Fig 1:**
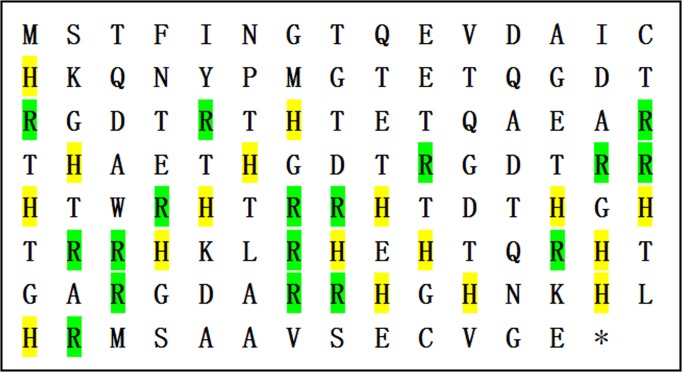
The amino acid sequence of rLj-RGD3. Histidines are highlighted in yellow; arginines are highlighted in green.

The cDNA of Lj-RGD3 (GenBank No. FJ416333) is 354 bp in length according to sequence analysis, and the translated protein sequence includes 2 cysteines, 17 histidines, 17 arginines, and 20 threonines in addition to 3 RGD motifs (Fig 1).

**Fig 2:**
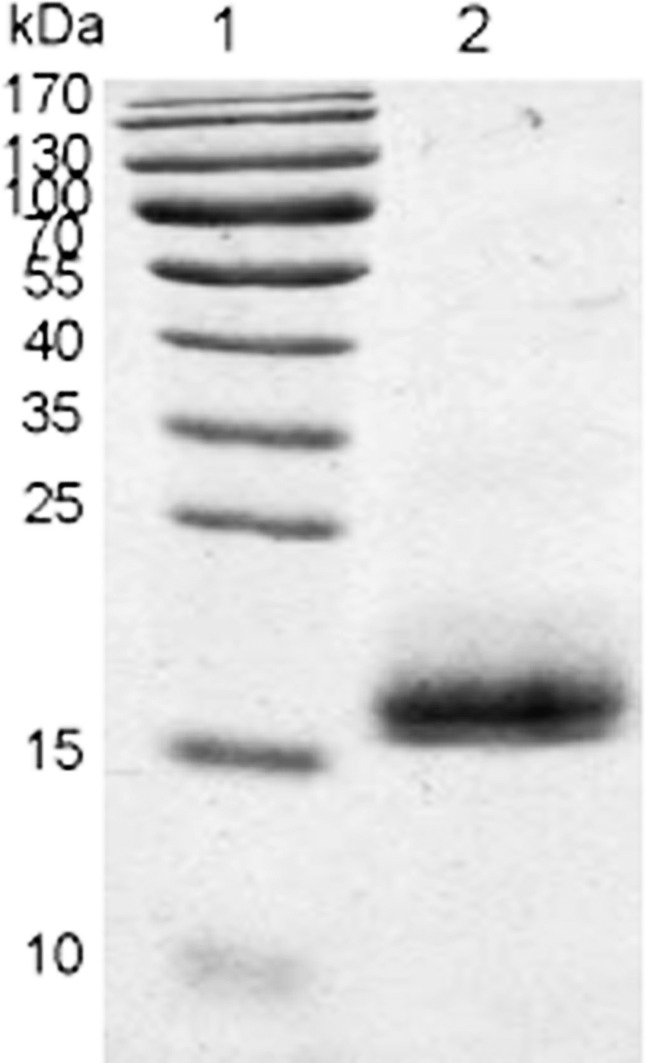
Tricine SDS-PAGE results showing purified rLj-RGD3. Lane 1: protein marker; Lane 2: a 14.5 kDa band indicating purified rLj-RGD3.

rLj-RGD3 was expressed as a soluble fusion protein in BL21 *E*. *coli* cells. rLj-RGD3 is a small protein in terms of molecular weight, and we identified it by attaching a his-tag to its C-terminus. The sequence was confirmed using NH_2_-terminal amino acid sequencing. The modified rLj-RGD3 protein migrated as a single band on SDS-PAGE gels at a position corresponding to approximately 17 kDa (Fig 2).

### Neurological evaluation

**Fig 3:**
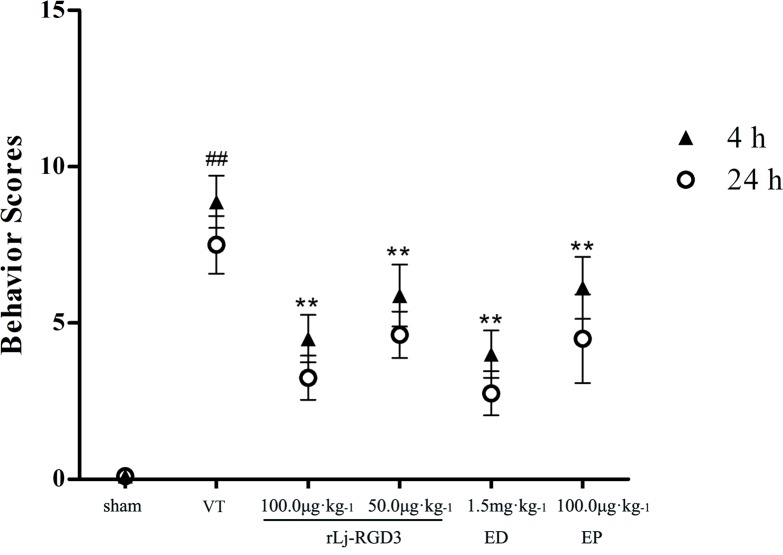
The effect of rLj-RGD3 on neurological deficit scores in focal cerebral ischemia/reperfusion rats. Neurological evaluations were used to assess rat behaviors after middle cerebral artery occlusion and reperfusion. The scores in the rLj-RGD3 groups were significantly lower (by 4 and 3) than that in the VT group (**p <0.01). rLj-RGD3 improved nerve functional deficits. ^##^*p* <0.01 *vs*. the sham group. The data are shown as the means ± SD, n = 6.

After completing neurological evaluations, we found no significant difference between the 25 μg·kg^-1^ group and the vehicle-treated (VT) group (S1 Fig). The results for the low-dose group (25 μg·kg^-1^) are therefore not shown. Neurological deficit scores are shown in Fig 3. The scores were significantly higher in the VT group than in the sham group. Treatment with rLj-RGD3 at a dose of 100.0 μg.kg^-1^ resulted in scores that were approximately 45% lower than those in the VT group. Moreover, the scores for the high-dose group were markedly lower than those for the rats that were treated with the drug Eptifibatide (*p*<0.05). In addition, there was no significant difference in scores between the high-dose RGD-containing peptide group and the Edaravone group. The results of further experiments showed that rLj-RGD3 significantly reduced reperfusion damage when the focal cerebral ischemia/reperfusion rats were injected with rLj-RGD3 at 5 hours after reperfusion (S3 Fig). However, the effect of administering the drug at 5 hours after reperfusion was less obvious than the effect of administration after 10 min. The neurological functions of the rats at 3 and 7 days after ischemia are described in S5 Fig As shown in S5 Fig, at 3 and 7 days after ischemia, the scores for neurological functions in the rats in the rLj-RGD3 administration groups were lower than those in the VT group.

### Infarct size and general histology

**Fig 4:**
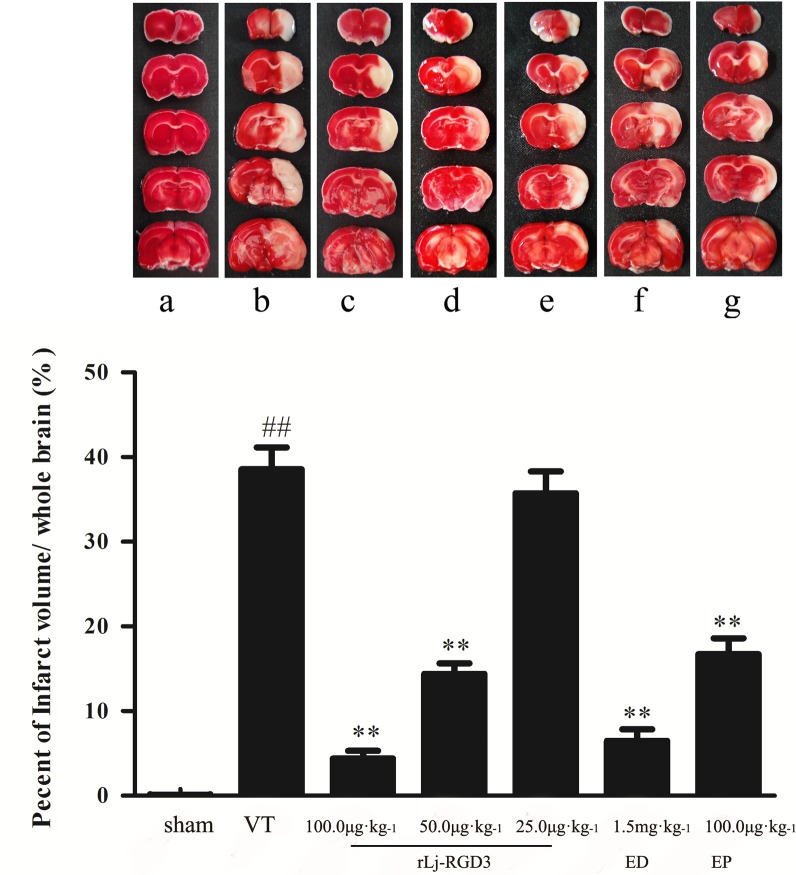
The effect of rLj-RGD3 on infarct volume in the brains of focal cerebral ischemia/reperfusion rats. a, b, c, d, e, f and g in panel A represent the sham group, the VT group, the rLj-RGD3 100.0 μg·kg^-1^ group, the rLj-RGD3 50.0 μg·kg^-1^ group, the rLj-RGD3 25.0 μg·kg^-1^ group, the Edaravone 1.5 mg·kg^-1^ group, and the Eptifibatide 100.0 μg·kg^-1^ group, respectively. TTC is a fat-soluble, light-sensitive complex. Normal tissue becomes red when dehydrogenase reacts with TTC. In contrast, ischemic tissue appears white because it lacks dehydrogenase. The greatest infarct volumes (approximately 40%) were observed in the VT group, and the infarct volumes were approximately 35% lower in the high-dose rLj-RGD3 group (**p <0.01 *vs*. the VT group). The data are shown as the means ± SD, n = 6.

**Fig 5:**
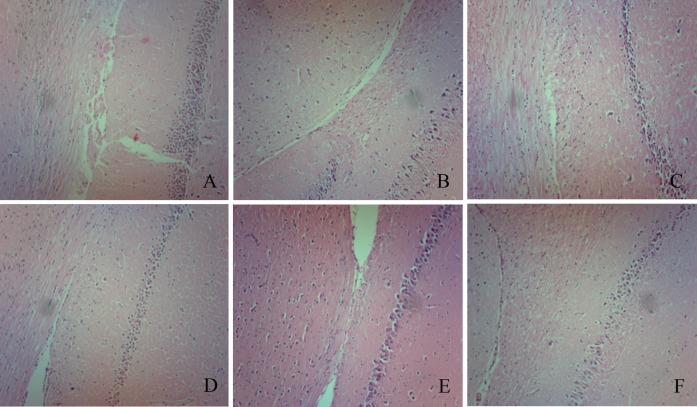
Pathology of focal cerebral ischemia/reperfusion rats (x100.0). A, B, C, D, E, and F represent the sham group, the VT group, the rLj-RGD3 100.0 μg·kg^-1^ group, the rLj-RGD3 50.0 μg·kg^-1^ group, the Edaravone 1.5 mg·kg^-1^ group, and the Eptifibatide 100.0 μg·kg^-1^ group, respectively. Tissue pathology was observed using H&E staining. No edema, hyperemia, or other abnormal morphological characteristics were observed in the sham group. The cone cell array and cell boundary are clearly visible. In contrast, the ischemic group presented an edematous morphology with vacuolated architecture and pyknotic nuclei following H&E staining. In addition, the VT group rat brains showed obvious cone cell disarrangement, and their cell boundaries were not clear. Adverse effects were ameliorated in each treatment group.

The highest infarct volumes (42.7%) were observed in the ischemic group (Fig 4A). However, rLj-RGD3 administration after reperfusion resulted in a significant reduction in infarct volume (a decrease of approximately 35%; Fig 4B), and the infarct volumes in the 25 μg·kg^-1^ group were not significantly different than those in the VT group (S2 Fig). The results of further experiments showed that rLj-RGD3 also significantly reduced infarct sizes in the focal cerebral ischemia/reperfusion rats that were injected with rLj-RGD3 at 5 hours after reperfusion (S4 Fig). However, the effect of drug administration at 5 hours after reperfusion was less obvious than that produced when the drug was administered at 10 min. The infarct size was obviously improved after rLj-RGD3 was administered for 3 and 7 days (S6 Fig). These results suggest that rLj-RGD3 is a genuinely effective drug. In addition, there was significant improvement in the behavioral outputs of the MCAO rats (p <0.01) compared to the outputs of the controls. The brains of the rats in the ischemic group exhibited an edematous morphology with vacuolated architecture and pyknotic nuclei in H&E staining, and this condition was effectively ameliorated by administering rLj-RGD3 (Fig 5).

### Neuronal apoptosis

**Fig 6:**
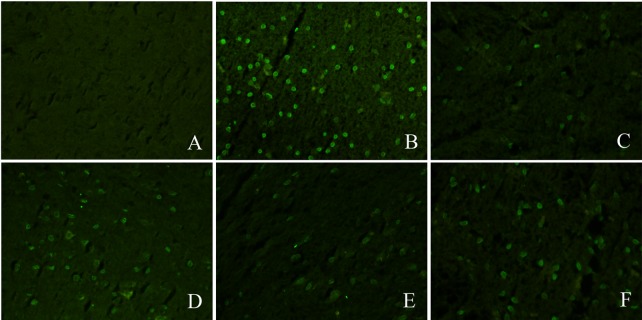
Neuronal apoptosis in the brains of focal cerebral ischemia/reperfusion rats as shown by TUNEL assay. A, B, C, D, E, and F represent the sham group, the VT group, the rLj-RGD3 100.0 μg·kg^-1^ group, the rLj-RGD3 50.0 μg·kg^-1^ group, the Edaravone 1.5 mg·kg^-1^ group, and the Eptifibatide 100.0 μg·kg^-1^ group, respectively. Neuronal apoptosis was not observed in the sham group. In contrast, a significant amount of neuronal apoptosis was observed in the VT group. Neuronal apoptosis was lower in all drug administration groups than in the VT group. Moreover, neuronal apoptosis was markedly lower in the high-dose group (rLj-RGD3 100.0 μg·kg^-1^) than in the other pretreatment groups.

Neuronal apoptosis was observed in the VT group. Compared to the ischemic group, there was a robust change in the amount of neuronal apoptosis in the rLj-RGD3 high-dose group (100.0 μg·kg^-1^). rLj-RGD3 significantly decreased the number of apoptotic neurons (Fig 6). rLj-RGD3 (high dose) clearly inhibited apoptosis in all of the pretreatment groups. These results demonstrate that rLj-RGD3 inhibits neuronal apoptosis in rat brain tissues.

### Western blotting and RT-PCR analysis

**Fig 7:**
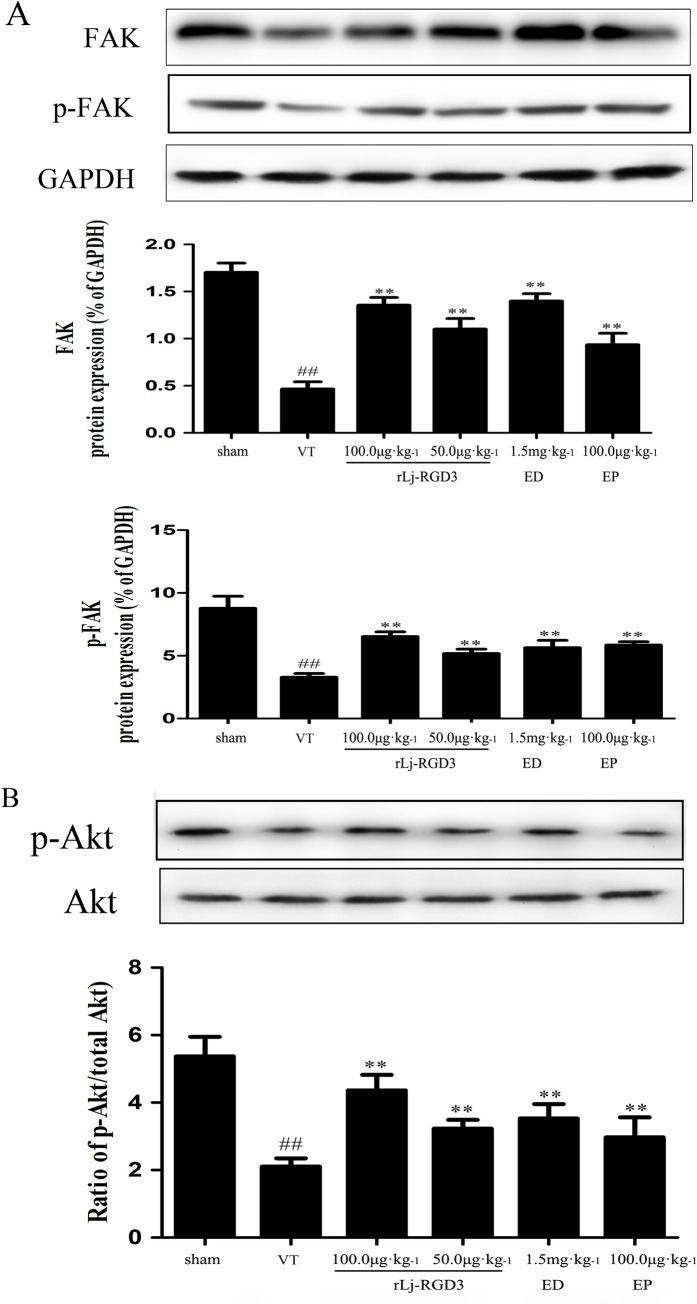
Expression of FAK proteins and the levels of p-FAK and p-Akt in focal cerebral ischemia/reperfusion rats as assayed using western blot analysis. The protein bands shown in the figure were quantified after the bands were scanned and normalized to GAPDH bands. The results are presented as the mean ± SD of three independent experiments. The level of each protein was markedly lower in the VT group rats (^##^*p* <0.01 *vs*. the sham group), and the expression of p-FAK and p-Akt were higher in the rLj-RGD3 rats (** *p*<0.01 *vs*. the VT group) and the positive control drug groups. The high-dose rLj-RGD3 group (100.0 μg·kg^-1^) exhibited significantly higher levels of FAK protein expression. ^##^p <0.01 *vs*. the sham group. The data are shown as the means ± SD, n = 6.

**Fig 8:**
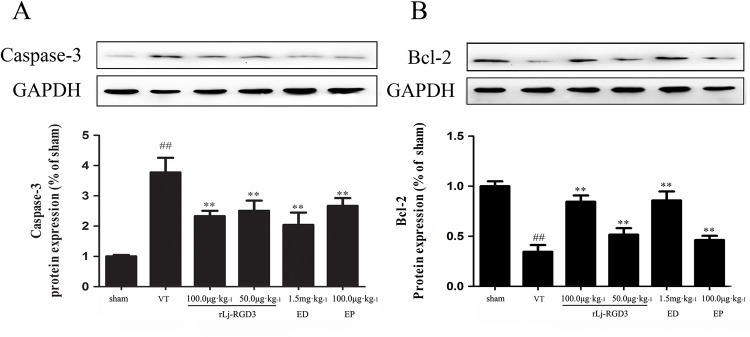
The effects of rLj-RGD3 on caspase-3 and Bcl-2 protein expression in focal cerebral ischemia/reperfusion rats as assayed using western blot analysis. The expression of caspase-3 protein was significantly higher in the VT group than in the sham group (^##^*p* <0.01). The rLj-RGD3 groups exhibited markedly lower levels of caspase-3 expression (**p <0.01 *vs*. the VT group). The anti-apoptosis gene Bcl-2 was markedly up-regulated in the rLj-RGD3 treatment groups, which demonstrated that rLj-RGD3 inhibits apoptosis. The data are shown as the means ± SD. For the western blotting analysis, one-way ANOVA was used to compare protein expression levels between the ischemia and sham groups.

**Fig 9:**
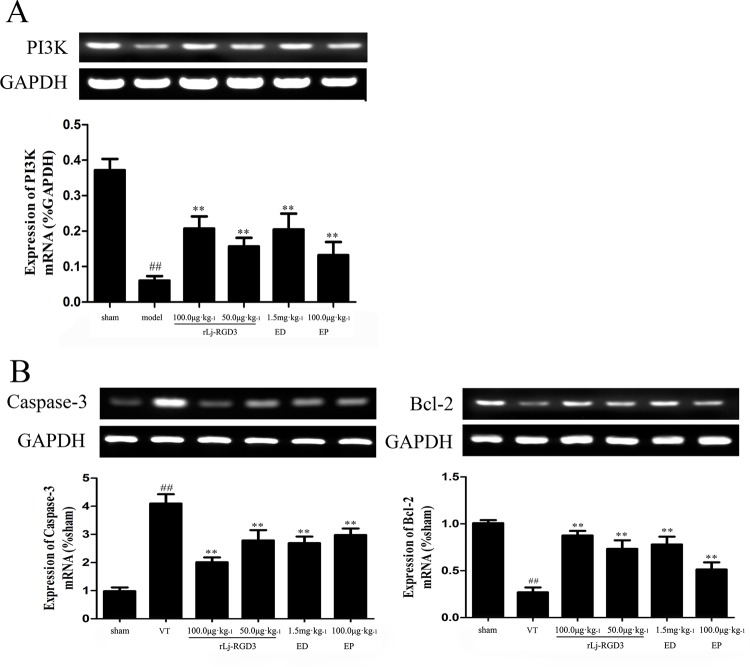
PI3K, caspase-3 and Bcl-2 mRNA levels detected using RT-PCR analysis in focal cerebral ischemia/reperfusion rats. PI3K and Bcl-2 mRNA expression in the ischemic cortexes of the cerebral ischemia/reperfusion rats was higher in the rLj-RGD3 groups than in the VT group. The level of caspase-3 mRNA was significantly lower in the rLj-RGD3 groups. rLj-RGD3 up-regulates PI3K and Bcl-2 mRNA levels and down-regulates caspase-3 mRNA expression. We therefore conclude that rLj-RGD3 is able to activate the PI3K/AKT anti-apoptosis pathway. The drug Eptifibatide resulted in expression levels that were less significantly higher than those in the rLj-RGD3 100.0 μg·kg^-1^ group (p <0.05). ^##^ *p*<0.01 *vs*. the sham group, **p <0.01 *vs*. the VT group. The data are shown as the means ± SD. For RT-PCR, one-way ANOVA was used to compare DNA bands between groups after ischemia.

Western blotting of tissue protein showed that FAK, p-FAK, p-Akt, and Bcl-2 were up-regulated in the ischemic group that was pre-treated with rLj-RGD3 (Figs 7 and 8B). As shown in Figs 8 and 9, rLj-RGD3 reduced the expression of caspase-3 (Figs 8A and 9B) at the protein and mRNA levels. These results suggest that rLj-RGD3 can salvage neurons in the ischemic cortex by activating the integrin-PI3K/Akt pathway to limit ischemic cell apoptosis.

**Fig 10:**
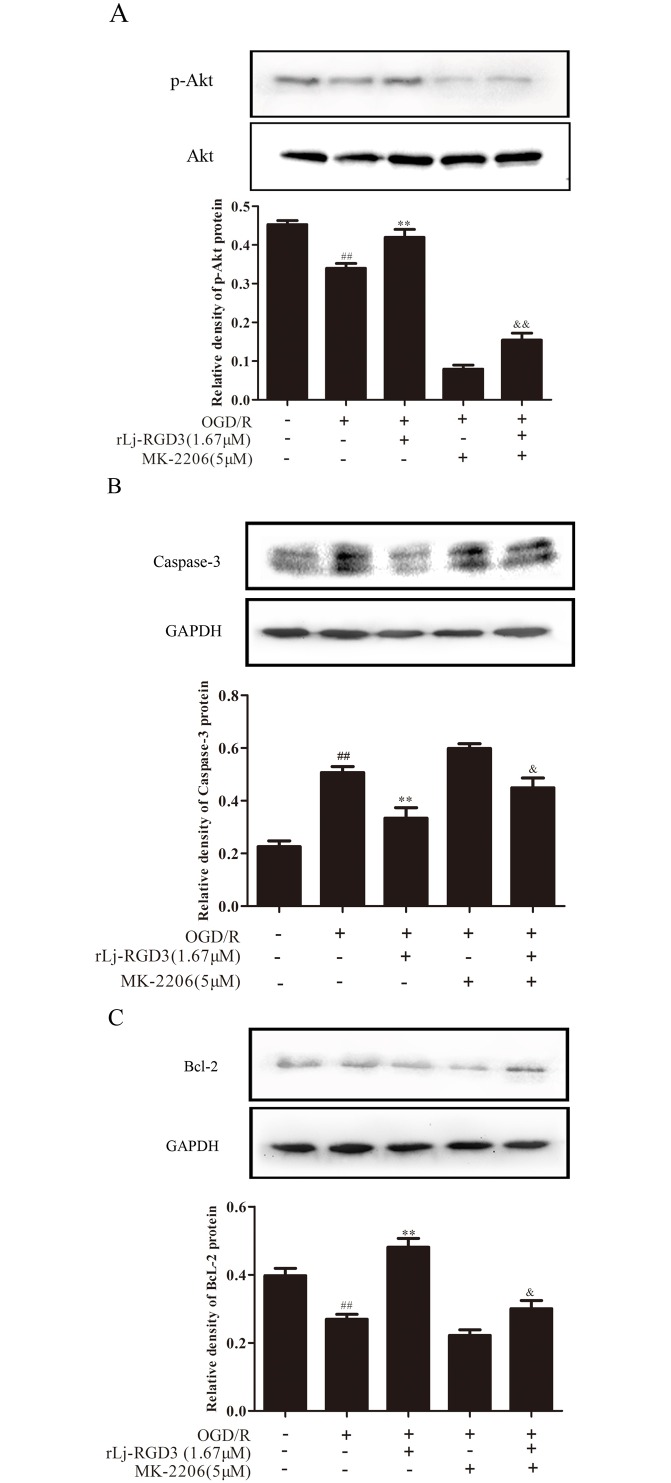
The role of PI3K/Akt signaling in the neuroprotective effects of rLj-RGD3 against OGD/R-induced PC12 cells apoptosis. P-Akt, caspase-3 and Bcl-2 expression levels in PC12 cells were assessed by western blotting. Our results show that OGD/R significantly inhibited Akt phosphorylation, but pretreatment with rLj-RGD3 attenuated the effects of OGD/R, indicating that rLj-RGD3 activates the PI3K/Akt pathway. MK-2206 significantly inhibited the effects of rLj-RGD3 treatment. The data are shown as the means ± SD, n = 3. ^##^ *p*<0.01 *vs*. the control group, **p <0.01 *vs*. the OGD/R group, ^&^ *p*<0.05 *vs*. the rLj-RGD3 group, ^&&^ *p*<0.01 *vs*. the rLj-RGD3 group.

To further investigate the role of PI3K/Akt signaling in the neuroprotective effects of rLj-RGD3 against OGD/R-induced apoptosis, PC12 cells were treated with 5 μM of the Akt inhibitor MK-2206. We evaluated Akt activation by western blot analysis. Our results show that OGD/R significantly inhibited Akt phosphorylation, but pretreatment with rLj-RGD3 attenuated the effects of OGD/R, indicating that rLj-RGD3 activates the PI3K/Akt pathway (Fig 10). MK-2206 significantly inhibited the effects of the rLj-RGD3 treatment, providing additional evidence for the role of PI3K/Akt signaling in the protective effects of rLj-RGD3.

Caspase-3 proteases are the major initiators of apoptosis. Activation of PI3K/Akt directly and indirectly induces the phosphorylation of caspase-3, promoting its interactions with other proteins and inhibiting apoptotic activity. As shown in Fig 10B, the protein levels of caspase-3 were significantly increased by OGD/R treatment compared with the control group. However, western blotting results showed that rLj-RGD3 decreased the levels of caspase-3 in OGD/R-treated cells. The Akt inhibitor MK-2206 attenuated the effects of rLj-RGD3, indicating that rLj-RGD3 inhibits caspase-3 expression through the activation of the PI3K/Akt signaling pathway.

Because PI3K/Akt pathway activation is known to up-regulate Bcl-2 and prevent apoptosis, we next evaluated the effects of rLj-RGD3 on Bcl-2 expression. As shown in Fig 10C, rLj-RGD3 attenuated the OGD/R-induced decrease in Bcl-2 protein levels, but the effect of rLj-RGD3 on Bcl-2 was diminished by MK-2206. This result provides further evidence for the role of PI3K/Akt signaling in the antiapoptotic effects of rLj-RGD3.

## Discussion

The protective effect of rLj-RGD3 was detected at the whole brain, cell and molecular levels in this study, which examined brain injuries induced by MCAO/RF. Neurological damage caused by cerebral ischemia/reperfusion in rats was significantly inhibited by rLj-RGD3, as revealed by behavioral scores. We used TUNEL staining to demonstrate that rLj-RGD3 inhibits the neuronal apoptosis that is caused by cerebral ischemia/reperfusion. The protective effect of rLj-RGD3 was demonstrated at the molecular level using western blot analysis and RT-PCR. Overall, we demonstrated that administering recombinant rLj-RGD3 following cerebral ischemia/reperfusion injury has a protective effect and partially inhibits nerve cell apoptosis.

RGD peptides compete with integrin to bind to extracellular matrix proteins, thereby affecting downstream pathways. The αvβ3 receptor features an RGD sequence (Arg-Gly-Asp). We previously showed that RGD-containing peptides inhibit platelet aggregation [24], angiogenesis [25,26], and neoplasm metastasis [27]. In this study, we demonstrated that rLj-RGD3 protects against cerebral ischemia/reperfusion damage.

Integrins function through two types of signal transduction pathways: intracellular signaling (inside-out signaling) and extracellular signaling (outside-in signaling). The former involves a high-affinity interaction between integrin receptors and their ligands that causes changes inside the cell. Integrin then binds to its secondary receptor and to ECM components, and receptors on the cell surface subsequently integrate into clusters that are induced by focal adhesion plaques. Lemons and others have revealed that integrin is closely involved in the repair of nervous system injuries [28]. Thus, focal adhesion kinase (FAK) might promote cell proliferation and inhibit apoptosis [29]. Integrins play a role in the activation of FAK, which promotes neuron growth. Recent research has suggested that an FAK-associated integrin signaling pathway might play an important role in nerve cell regeneration [30]. In our experiments, FAK expression was significantly lower in the VT groups than in the sham-operated group, and the rLj-RGD3 MCAO/RF group showed a notable increase in the expression of FAK and p-FAK, which resulted in a protective effect in the brain. Moreover, the effect of rLj-RGD3 was more pronounced than the effect of the similar drug Eptifibatide.

The PI3K family is complex. In normal cells, P13K activity is affected by several strictly regulated mechanisms. In resting cells, P85-P110 compounds that are commonly found in the cytoplasm require an appropriate activation signal. In the rat brain, integrins that are present on the surface of neuronal cells combine with RGD peptides to activate PI3K, thus generating the second messenger PIPa at the cell membrane. PIPa induces PDKl phosphorylation (at Ser308) and thereby activates Akt. Akt then directly phosphorylates several transcription factors, thus inhibiting the expression of apoptosis-related genes and enhancing the expression of anti-apoptosis genes to promote cell survival. Akt might also directly inhibit the activation of caspase-9. The activity of pro-caspase-9 is eliminated after it is phosphorylated by Akt, which interrupts downstream caspase-3 expression. Cell apoptosis is consequently decreased. In the areas of cell necrosis that occur near ischemia, apoptosis occurs mainly in the ischemic penumbra [31]. Delayed neuronal death in cerebral ischemia is dominated by apoptosis, and the extent of apoptosis determines the final infarct size [32]. Current research on apoptosis-related genes and proteins has not provided great insight into this issue. Neuronal apoptosis after cerebral ischemia/reperfusion might be associated with apoptosis-related protein factors and cytokines, such as cytochrome c, and caspases. Caspase-3 and mammalian cell protease are key players during apoptosis. Caspase-3 is inactive under normal conditions and is activated only during apoptosis [33]. Caspase-3 participates in brain ischemic damage, and caspase-3 inhibitor therapy might reduce infarct volumes in rats and thereby lessen the severity of neurologic impairments.

Bcl-2 is currently the best-studied gene that is known to inhibit apoptosis. Several studies have shown that Bcl-2 inhibits apoptosis to some extent following cerebral ischemia/reperfusion injury [34,35]. In our study, surviving nerve cells exhibited increased Bcl-2 expression, while the expression of this protein was decreased in apoptotic cells. Pretreatment with rLj-RGD3 significantly increased Bcl-2 expression, suggesting a mechanism by which rLj-RGD3 might inhibit apoptosis.

In conclusion, our findings confirm that activation of the PI3K/Akt anti-apoptotic pathway by rLj-RGD3 is integrin-dependent. We also showed that the curative effect of rLj-RGD3 is far superior to that of the similar drug Eptifibatide. Edaravone is currently used as a clinical cerebral protective agent, and our results show that rLj-RGD3 performed better than this agent in preclinical experiments. We also demonstrated that rLj-RGD3 exerts a protective effect following cerebral ischemia/reperfusion injury. rLj-RGD3 distinctly improves behavioral problems in postoperative rats, reduces infarct volume due to MCAO/RF damage, and inhibits neuronal apoptosis. These results provide a theoretical foundation and an experimental basis for developing rLj-RGD3 as a drug for the treatment of ischemic cerebral vascular diseases.

## Supporting Information

S1 Fig The effect of rLj-RGD3 on neurological deficit scores in focal cerebral ischemia/reperfusion rats. As the figure shows, there was no significant difference between the VT group and the 25 μg·kg^-1^ rLj-RGD3 group. ^##^p<0.01, vehicle-treated (VT) group *vs*. sham group; **p<0.01, 100 μg·kg^-1^ *vs*. VT group; *p<0.05, 50 μg·kg^-1^ *vs*. VT group.
(TIF)

S2 Fig The effect of rLj-RGD3 on infarct volume in the brains of focal cerebral ischemia/reperfusion rats. a: sham group, b: vehicle-treated (VT) group, c: 100 μg·kg^-1^ rLj-RGD3 group, d: 50 μg·kg^-1^ rLj-RGD3 group, and e: 25 μg·kg^-1^ rLj-RGD3 group. As the figure shows, there was no significant difference between the VT group and the 25 μg·kg^-1^ rLj-RGD3 group. ^##^p<0.01, vehicle-treated (VT) group *vs*. sham group; **p<0.01, 100 μg·kg^-1^ *vs*. VT group.
(TIF)

S3 Fig The effect of rLj-RGD3 on neurological deficit scores in focal cerebral ischemia/reperfusion rats that were injected with rLj-RGD3 at 5 hours after reperfusion. ^##^p<0.01, vehicle-treated (VT) group *vs*. sham group; *p<0.05, 100 μg·kg^-1^ *vs*. VT group.
(TIF)

S4 Fig The effect of rLj-RGD3 on infarct volume in the brains of focal cerebral ischemia/reperfusion rats that were injected with rLj-RGD3 at 5 hours after reperfusion. a: sham group, b: vehicle-treated (VT) group, c: 100 μg·kg^-1^ rLj-RGD3 group. ^##^p<0.01, vehicle-treated (VT) group *vs*. sham group; **p<0.01, 100 μg·kg^-1^ *vs*. VT group.
(TIF)

S5 Fig The effect of rLj-RGD3 on neurological deficit scores at 3 and 7 days after ischemia induction. The results show that the neurological deficit scores were clearly improved after rLj-RGD3 was administered for 3 and 7 days. ^##^p<0.01, vehicle-treated (VT) group *vs*. sham group; **p<0.01, 100 μg·kg^-1^ *vs*. VT group; *p<0.05, 100 μg·kg^-1^ *vs*. VT group.
(TIF)

S6 Fig The effect of rLj-RGD3 on infarct volume in the brains of focal cerebral ischemia and reperfusion rats at 3 and 7 days after ischemia induction. A: TTC staining of a brain at 3 days after ischemia. B: TTC staining of a brain at 7 days after ischemia. C: The infarct volumes at 3 and 7 days after ischemia. ^##^p<0.01, vehicle-treated (VT) group *vs*. sham group; **p<0.01, 100 μg·kg^-1^ and 50 μg·kg^-1^ *vs*. VT group.
(TIF)
