# Supplementary figures and images for: rLj-RGD3, a Novel Recombinant Toxin Protein from Lampetra japonica, Protects against Cerebral Reperfusion Injury Following Middle Cerebral Artery Occlusion Involving the Integrin-PI3K/Akt Pathway in Rats
Source: PLoS One. 2016 Oct 21;11(10):e0165093. doi: 10.1371/journal.pone.0165093 (PMC5074578; doi:10.1371/journal.pone.0165093)

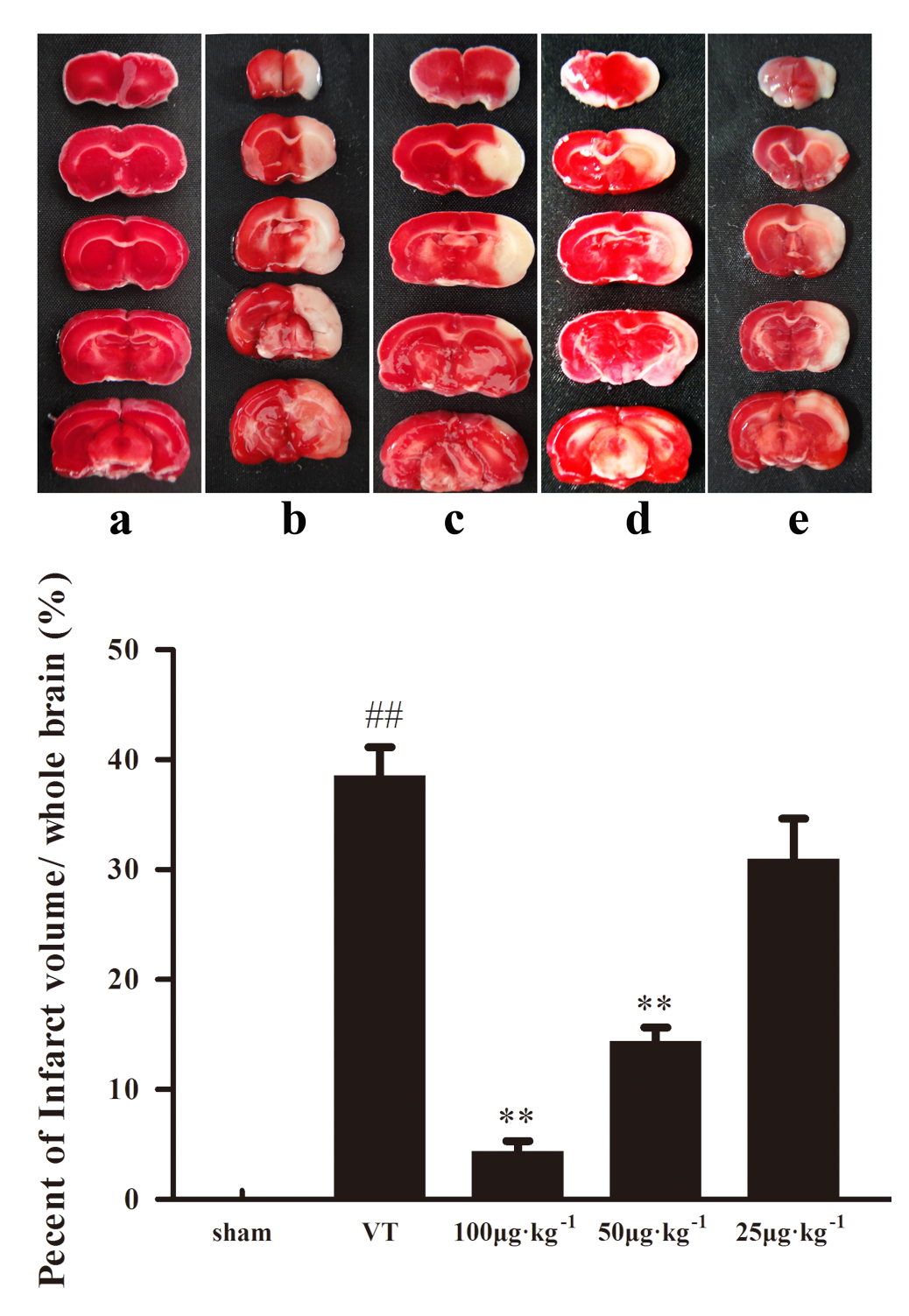

Supplement: S1 Fig — As the figure shows, there was no significant difference between the VT group and the 25 μg·kg-1 rLj-RGD3 group. ##p<0.01, vehicle-treated (VT) group vs. sham group; **p<0.01, 100 μg·kg-1 vs. VT group; *p<0.05, 50 μg·kg-1 vs. VT group. (TIF) [file pone.0165093.s001.tif]

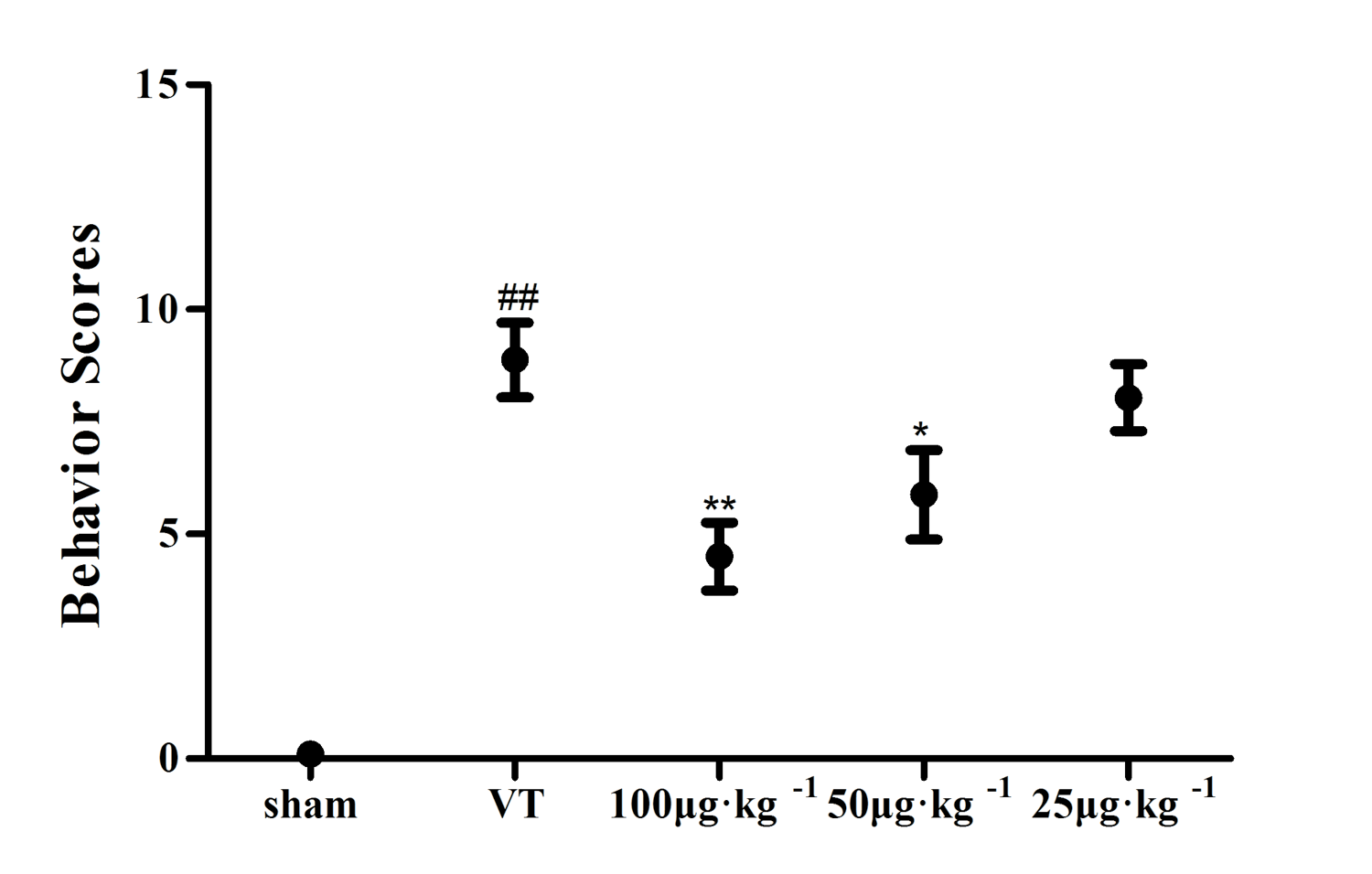

Supplement: S2 Fig — a: sham group, b: vehicle-treated (VT) group, c: 100 μg·kg-1 rLj-RGD3 group, d: 50 μg·kg-1 rLj-RGD3 group, and e: 25 μg·kg-1 rLj-RGD3 group. As the figure shows, there was no significant difference between the VT group and the 25 μg·kg-1 rLj-RGD3 group. ##p<0.01, vehicle-treated (VT) group vs. sham group; **p<0.01, 100 μg·kg-1 vs. VT group. (TIF) [file pone.0165093.s002.tif]

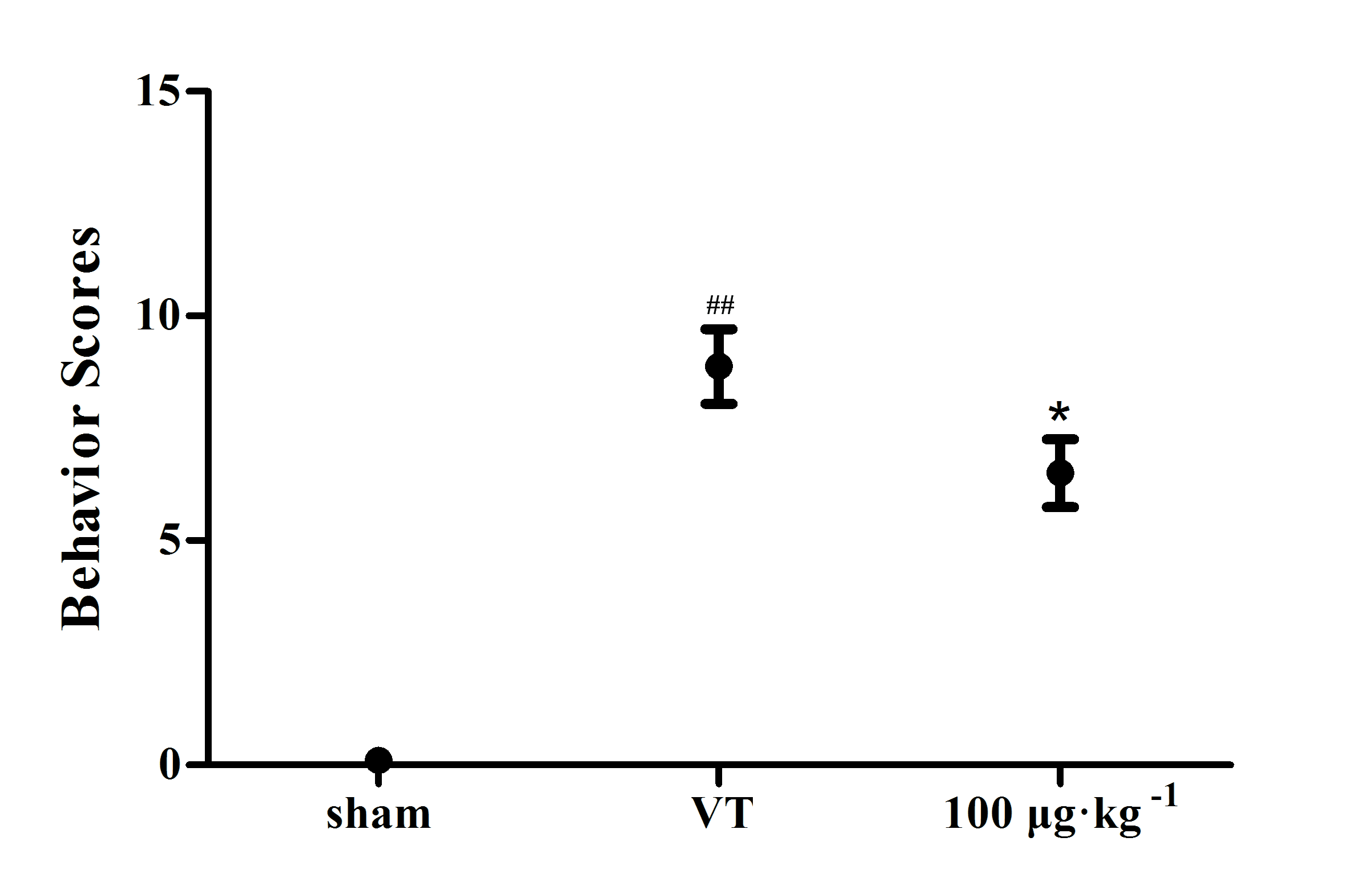

Supplement: S3 Fig — ##p<0.01, vehicle-treated (VT) group vs. sham group; *p<0.05, 100 μg·kg-1 vs. VT group. (TIF) [file pone.0165093.s003.tif]

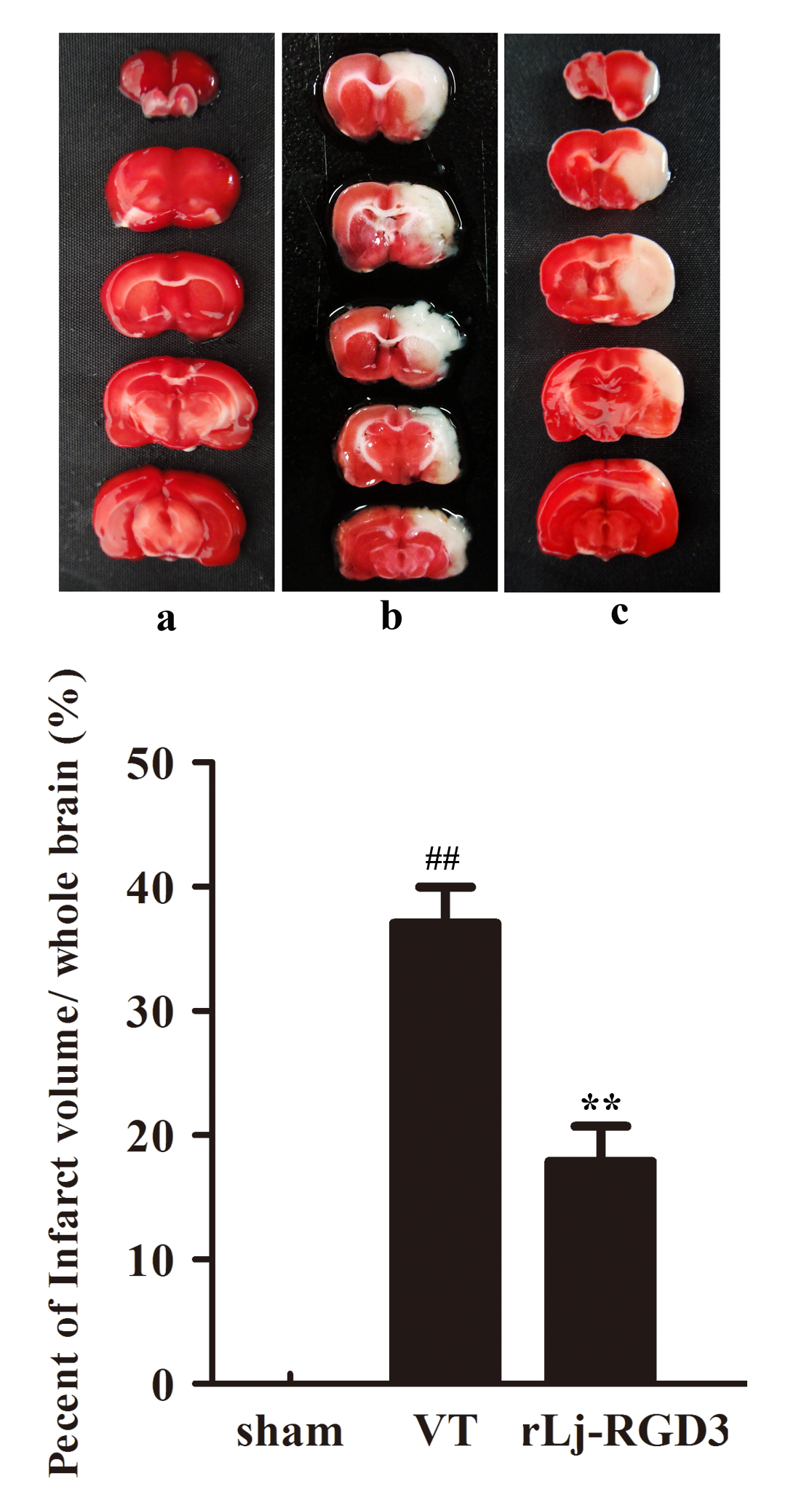

Supplement: S4 Fig — a: sham group, b: vehicle-treated (VT) group, c: 100 μg·kg-1 rLj-RGD3 group. ##p<0.01, vehicle-treated (VT) group vs. sham group; **p<0.01, 100 μg·kg-1 vs. VT group. (TIF) [file pone.0165093.s004.tif]

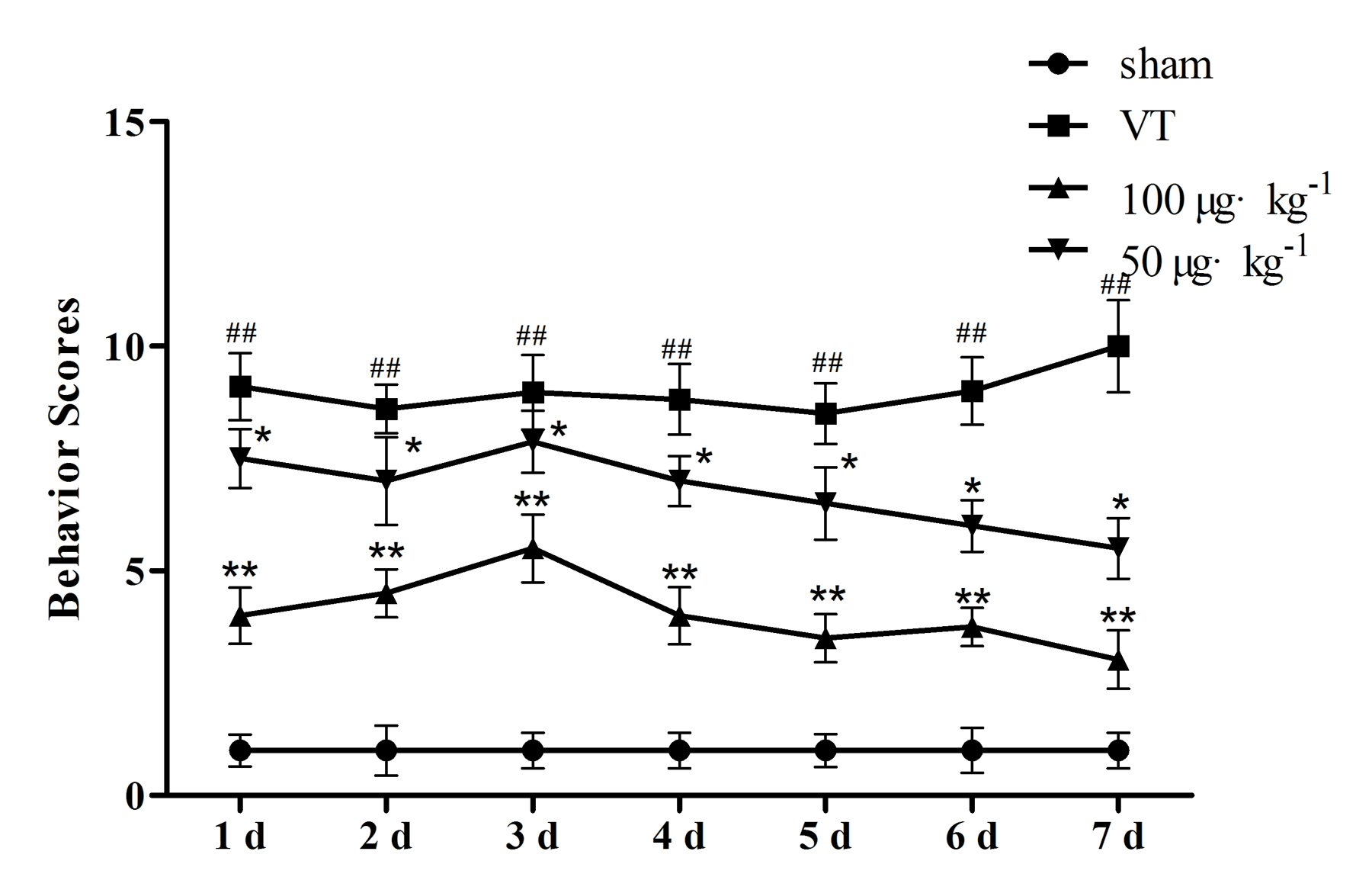

Supplement: S5 Fig — The results show that the neurological deficit scores were clearly improved after rLj-RGD3 was administered for 3 and 7 days. ##p<0.01, vehicle-treated (VT) group vs. sham group; **p<0.01, 100 μg·kg-1 vs. VT group; *p<0.05, 100 μg·kg-1 vs. VT group. (TIF) [file pone.0165093.s005.tif]

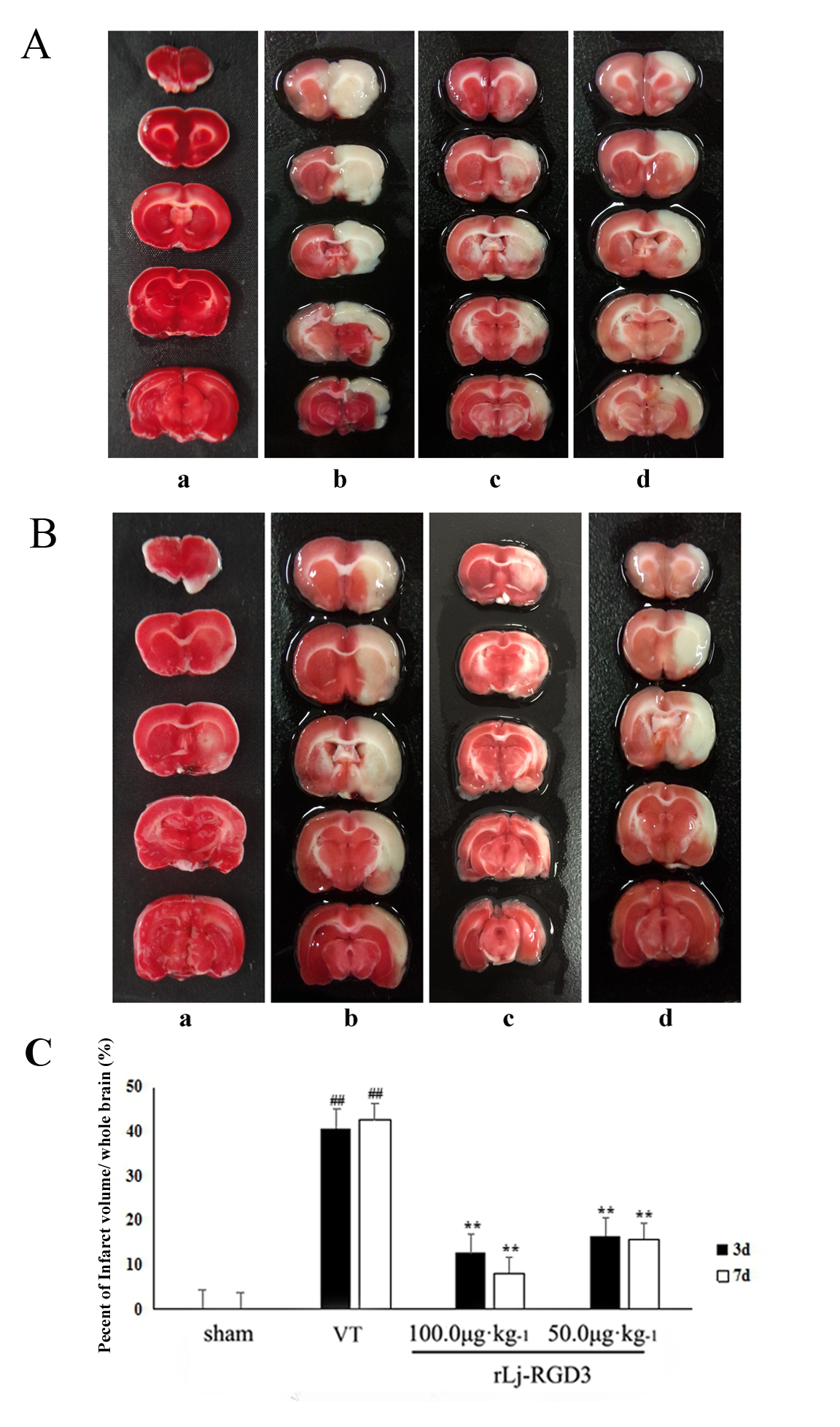

Supplement: S6 Fig — A: TTC staining of a brain at 3 days after ischemia. B: TTC staining of a brain at 7 days after ischemia. C: The infarct volumes at 3 and 7 days after ischemia. ##p<0.01, vehicle-treated (VT) group vs. sham group; **p<0.01, 100 μg·kg-1 and 50 μg·kg-1 vs. VT group. (TIF) [file pone.0165093.s006.tif]
